# Alignment of Canada's COVID-19 policy response with barriers and facilitators for coping reported by caregivers of youth with developmental delays, disorders, and disabilities

**DOI:** 10.3389/fresc.2024.1308062

**Published:** 2024-03-25

**Authors:** Anna Katalifos, Mayada Elsabbagh, Afiqah Yusuf, Sakiko Yamaguchi, Julie Scorah, Nicola Wright, Mandy Steiman, Andy Shih, Keiko Shikako

**Affiliations:** ^1^ Azrieli Centre for Autism Research, Montreal Neurological Hospital-Institute, McGill University Health Centre, Montreal, QC, Canada; ^2^ Department of Neurology and Neurosurgery, Faculty of Medicine and Health Sciences, McGill University, Montreal, QC, Canada; ^3^ School of Physical and Occupational Therapy, Faculty of Medicine and Health Sciences, McGill University, Montreal, QC, Canada; ^4^ Department of Psychology, Faculty of Health, Psychology, and Social Care, Manchester Metropolitan University, Manchester, United Kingdom; ^5^ Scientific Affairs, Autism Speaks, New York, NY, United States

**Keywords:** developmental disability, coping, COVID-19 pandemic, caregiver, policy & disabilities

## Abstract

**Introduction:**

The UNICEF-WHO Global Report on Developmental Delays, Disorders, and Disabilities is an ongoing initiative aimed at increasing awareness, compiling data, providing guidance on strengthening health systems, and engaging country-level partners. Data from its caregiver survey assessing impacts of the COVID-19 pandemic showed that half of youths with developmental delays and disabilities (DDDs) and their caregivers struggled to cope, with a significant portion reporting a lack of supports and difficulty managing the worsening of the child's symptoms in isolation. Governments created service strategies supporting vulnerable groups. Little is known about the alignment between COVID-19 policies for persons with disabilities and their lived experiences. Contextualizing caregivers’ experiences can promote the development of tailored public supports for these families following a public health crisis.

**Methods:**

Online survey data were collected from June-July 2020, leading to a convenience sample of caregivers of youth with DDDs across Canada. Respondents answered two open-ended questions regarding challenges and coping strategies during the pandemic. We conducted a thematic analysis of responses using inductive coding on NVivo software. Overarching codes derived from the dataset were contextualized using an analysis of provincial policies published during the pandemic. Parallels with these policies supported the exploration of families’ and youths’ experiences during the same period.

**Results:**

Five hundred and seventy-six (*N* = 576) participants answered open-ended questions. Barriers to coping included family mental health issues, concerns about the youths’ regression, challenges in online schooling, limited play spaces, and managing physical health during quarantine. Environmental barriers encompassed deteriorating family finances, loss of public services, and a lack of accessible information and supports. In contrast, caregivers reported coping facilitators, such as family time, outdoor activities, and their child's resilience. Environmental facilitators included community resources, public financial supports, and access to telehealth services. Few COVID-19 policies effectively addressed caregiver-identified barriers, while some restrictions hindered access to facilitators.

**Conclusion:**

Prioritizing needs of families of youths with DDDs during public health emergencies can significantly impact their experiences and mental health. Enhancing financial benefits, providing telehealth services, and creating inclusive public play spaces are priority areas as we navigate the post-pandemic landscape.

## 1 Introduction

In March 2020, the World Health Organization (WHO) declared the novel coronavirus (COVID-19) outbreak a global pandemic. Many countries imposed various restrictive public health measures to mitigate the spread of the virus, including social distancing, mandatory quarantining for citizens at home (1), the closure or suspension of schools, daycares, health and social services, and in some cases, obligatory curfews (2). These public health measures were gradually lifted, and in some instances reinstated, depending on outbreak severity.

While many governments successfully slowed the spread of COVID-19 through the adoption of these regulations, scholarly findings indicate that children and youth with disabilities experienced negative impacts on their wellbeing along with limited or reduced access to services and supports (3, 4). Compelling evidence suggests that pre-existing vulnerabilities and inequities for many children and youth with developmental disabilities and disorders (DDDs) were amplified by the pandemic (5, 6). In many countries, policies were published and implemented at various levels of government to support vulnerable populations (7, 8). There is a dearth of research, however, surrounding the alignment between the needs and experiences of children and youth with disabilities, and this knowledge gap exists in Canada's COVID-19 policy context. Information is also limited on the alignment of public policies with international guidance such as those proposed by the WHO and the United Nations (UN).

### 1.1 Rights-based approaches in policymaking for developmental delays, disorders, and disabilities

Evidence suggests that policymaking for individuals with disabilities is most comprehensive when aligned with human-rights-based approaches (9). An example is the 2006 *United Nations Convention on the Rights of Persons with Disabilities* (UNCRPD), an international treaty ratified by 184 States Parties, including Canada. The UNCRPD provides signatories with a “code of implementation” to follow when drafting laws or administrative measures related to persons with disabilities, supporting the promotion of human rights while setting guidelines for abolishing discriminatory legislation (10). The Treaty advances the disability rights movement by shifting the paradigm of viewing persons with disabilities as “objects” of charity toward “subjects” with rights and agency as well as active members of their communities (10).

In Canada, public strategies to support persons with disabilities were implemented by federal, provincial, and territorial governments following the ratification of the UNCRPD. Current federal accessibility legislation includes the 2019 *Accessible Canada Act* (ACA), passed to remove barriers to inclusion for persons with disabilities within the federal sphere while preventing the emergence of new barriers (11). The ACA defines a disability as “any impairment, including a physical, mental, intellectual, cognitive, learning, communication or sensory impairment […] whether permanent, temporary, or episodic in nature, or evident or not, that, in interaction with a barrier, hinders a person's full and equal participation in society.” The ACA also establishes a framework for accessibility standards, setting the objective to achieve a barrier-free Canada by 2040 (11).

The Canadian federalist context renders some jurisdictions as federal responsibilities (e.g., citizenship, unemployment insurance, national defence) and others as provincial powers (e.g., health services, education, social welfare). Most daily essential services for children and youth with disabilities are of provincial responsibility, accessed through healthcare and educational institutions. The provinces of Ontario, Manitoba, Nova Scotia, and British Columbia have adopted specific accessibility legislation as of 2005 (12–15). Across jurisdictions, concrete public supports for these youths and their families are translated into financial benefits and income support programs, tax measures, community and caregiver support programs, housing programs, employment measures, educational programs, and subsidies for advocacy groups supporting persons with disabilities. Evidence suggests that expenditures and public service users in Canada who have at least one disability have continued to rise across most provinces since 2000 (16).

### 1.2 Children with developmental delays, disorders, and disabilities, and vulnerability in public health emergencies

Despite Canada's ratification of the UNCRPD and the implementation of numerous public policies supporting persons with disabilities, this population is still vulnerable to adversities in Canada. According to a 2018 report published by Statistics Canada, persons with more severe disabilities are at increased risk of living in poverty than their counterparts without disabilities or with milder disabilities (17). This same report found that, as disability severity level increased, the likelihood of being employed decreased, albeit that two in five of the individuals with a disability who were not employed and not currently in school had the potential to work (17). Moreover, academic research indicates that the needs of Canadian families of children and youth with disabilities are inadequately met by public supports, as they may face insufficient access to social activities and information regarding services available to them and more frequent interrupted service provision (18). Other obstacles regarding public service utilization for these youths and their families within high-income countries include inadequate insurance coverage, difficulty obtaining referrals to specialist healthcare providers, and a lack of care coordination and shared decision-making between service providers (18, 19).

Children are generally more susceptible to negative outcomes during disasters, and special protections are granted for children with disabilities, as enshrined by the UNCRPD and the UN Convention on the Rights of Children. Youth with DDDs are at higher risk for socioeconomic hardship and homelessness, poor nutrition, domestic and sexual abuse, higher levels of stress and mental health complications, and bullying (20, 21). Moreover, caregivers of youth with a neurodevelopmental disability have also been found to be increasingly likely to experience financial hardship, high levels of stress, and mental health complications (22, 23). Parent stress can be characterized as psychological symptoms of distress experienced by parents as a result of aversive responses to parental obligations (24, 25), and has been linked to negative impacts on the child's wellbeing (26). Causes of stress in parents of children with disabilities include concerns about the child's symptoms, such as distressed behaviours (27), parents’ socioeconomic status (28), child sleep problems (29), and difficulties in access to services (30). In contrast, coping is defined as a “behavioural reaction to aversive situations” (31) and is associated with decreased stress levels and better child outcomes (31–33). While coping is an intrinsic mechanism, external circumstances can facilitate or hinder parents’ ability to cope, with direct consequences on their child and family well-being.

Evidence suggests that the vulnerabilities, inequities, and gaps in services and supports for children with DDDs are further exacerbated in the context of disastrous events. Disastrous events have been defined differently across the existing literature. For the purpose of this study, a disastrous event is referred to as a hazard that has consequences regarding damages to livelihood, economic disruptions, and/or casualties that are too great for the affected area and for individuals to manage without supports (34). Following this description, the COVID-19 pandemic qualifies as a global disastrous event, in that it caused millions of deaths worldwide (35), significant disruptions to livelihood globally (36, 37), and long term negative impacts on world economies (38, 39).

### 1.3 The COVID-19 pandemic and developmental delays, disorders, and disabilities

The COVID-19 pandemic and disruptions to essential services posed significant challenges for individuals with DDDs. A recent scoping review by Taggart et al. sought to establish key learning points emerging from the literature regarding the experiences of persons with DDDs during the pandemic (40). This review revealed that policy responses in several high-income countries, despite prior ratification of the UNCRPD, fell short in safeguarding the human rights of individuals with DDDs. Issues included limited availability to personal protection materials, lack of plain-language information, essential service closures, and, disturbingly, compulsory covert “do not resuscitate” orders, among others (40). Findings from this scoping review stress the need for better inclusion of this population in emergency planning and responses for future pandemics and disasters.

Moreover, the impacts of public health measures adopted to mitigate the spread of the COVID-19 virus contributed to mental health challenges for children with DDDs, resulting from a lack of access to social networks and activities, restricted access to health supports, and tensions within family units (41–43). The pandemic was also challenging for caregivers of youth with DDDs in that they experienced higher stress levels and mental health complications than parents of neurotypical youth (44, 45) and of youth with intellectual disabilities or a visual or hearing impairment (46). Higher caregiver stress levels may have further exacerbated negative impacts of the pandemic on children with DDDs as they may have needed to rely on their caregivers more heavily. Other negative impacts of the pandemic on the wellbeing of children with DDDs as reported by their caregivers were reduced exercise and poorer sleep and diet quality (47).

Reported pandemic-related stressors in caregivers included changes in their children's routines, worrying about contracting the COVID-19 virus, and transitioning to online learning (48). One study found that over half of parents of youth perceived an increase in stress during the pandemic, notably related to the closure of child facilities and social distancing, with a subgroup of these parents reporting heightened depressive symptoms and anxiety (49). Parent stress is an important factor to consider in the context of the pandemic, as it has been found to impact the emotional regulation and lability/negativity of their children, with parent-perceived self-efficacy acting as a mediator (50). Moreover, early evidence from the pandemic indicates that many parents who experienced negative mental health consequences related to the pandemic did not access any online or phone psychiatric support (51). The wellbeing of these children may also be affected, as low parent self-efficacy has been linked to increased internalizing problems and negative emotionality in children when compared with caregivers with high parent self-efficacy (52).

### 1.4 The WHO Global Report Survey on Developmental Delays, Disorders, and Disabilities

Assessing the impact of the COVID-19 pandemic on youth with DDDs and their caregivers by considering their experiences helps to identify priority areas for service improvement. The *Global Report Survey on Developmental Delays, Disorders, and Disabilities*, henceforth the *Global Report Survey*, exists as an ongoing initiative led by the WHO, UNICEF, and Autism Speaks to describe experiences of caregivers of youth with DDDs worldwide (53). This project seeks to increase awareness, compile novel data, provide guidance to strengthen health systems, and to engage international partners. The development of the *Global Report Survey* began before the COVID-19 pandemic. Due to additional challenges faced by families of youth with DDDs during the initial months of the pandemic, objectives for the *Global Report Survey* were adjusted to reflect potential impacts. The aims of the *Global Report Survey* in Canada were thus adapted to “assess the impact of the pandemic on the health and wellbeing of caregivers and their children” and to “understand the patterns of help seeking access to services and supports prior to and during the pandemic” (53).

Canadian federal and provincial governments implemented public service strategies to address challenges faced by disabled youth and their families during the pandemic (8). However, evidence from the Canadian iteration of the *Global Report Survey* suggests that many caregivers of youth with DDDs reported difficulties with accessing information regarding services available during the pandemic and an overall worsening of children's symptoms related to their disability (53). Further findings from the *Global Report Survey* indicate that some youth with disabilities and their families may have faced more layers of vulnerability than others. A recent study using this data found that various sociodemographic characteristics of families of youth with DDDs affected their receipt of physical and mental health services during the pandemic (54). Caregiver-related factors that decreased the likelihood of receiving services were being a single parent, having low educational attainment, working less than full time, and having a yearly income lower than CAD$40,000. Child- and youth-related factors that decreased the likelihood of receiving services were male gender and older age (54).

Other findings from the *Global Report Survey* in Canada support the notion that, while many youths with DDDs experienced negative impacts because of the pandemic, a considerable minority displayed resilience. Resilience is defined as experiencing better-than-expected outcomes in the face of adversity (55). For individuals with some diagnoses, such as autism spectrum disorder, some risk factors for hindered resilience can be considered modifiable to improve resilience outcomes, namely enhanced parenting self-efficacy, outside of the context of a disastrous event (56). A latent class analysis of the Canadian *Global Report Survey* data found that parenting self-efficacy and support in accessing schooling were potentially modifiable factors related to resilience in children with a DDDs during the pandemic (57). This same analysis highlighted the need for tailored supports responding to different diagnoses through interventions fostering caregiver empowerment along with maintained access to schooling, health, and social services (57). Evidence suggests that some parents of youth with a DDD in other countries found establishing coping strategies useful in managing the impact of the pandemic (58). Strategies included structuring their days, using visual supports or new technologies for learning and leisure, and online contact with relatives and psychological supports (58).

While the COVID-19 pandemic presented negative impacts on Canadian families of youth with DDDs and exacerbated existing inequities, there remains a dearth of information regarding whether their needs aligned with public supports created during the same period, and particularly in relation to the UNCRPD. Considering factors that promote resilience in Canadian youths and their families is essential in improving their outcomes as we develop tailored supports for the transition out of the pandemic. This study aims to:

1. Describe barriers and facilitators related to coping identified by caregiver of youth with DDDs in Canada during the pandemic.
2. Contextualize the experiences of youth with DDDs and their caregivers in relation to Canadian COVID-19 policies for persons with disabilities to identify alignment and gaps.

A secondary objective is to inform public policy and services on the areas of need in recovering from the pandemic. Understanding families’ experiences during the pandemic can inform better integration of their needs and UNCRPD considerations into public policies and programs.

## 2 Materials and methods

### 2.1 Study design and participants

The data source for this study is qualitative data (open-ended responses) from the Canadian iteration of the WHO *Global Report Survey on Developmental Delays, Disorders, and Disabilities*. Questions for the *Global Report Survey* were developed based on COVID-19 UNICEF and WHO policy guidance recommendations for persons with disabilities, and the United Nations Washington Group Disability Statistics indicators (54, 59, 60). In Canada, question topics for the *Global Report Survey* included a set of questions related to the COVID-19 pandemic experiences and subsequent access to care supports, mental health impact, and coping (a total of 49 Likert-scale, multiple choice, and open-ended questions). The survey was distributed online through social media platforms along with mailing lists of partner organizations and individual collaborators, including parents and researchers within the team's network. This resulted in a non-random, convenience sample of caregivers of youth with DDDs. A cross-sectional design was used, and the survey was available in English and French. Data were collected from June 11 to July 21, 2020. Participants of the survey were identified as primary caregivers to a child, youth, or adult with a DDD. Each participant was offered CAD$15 for their participation in the survey, and written informed consent was obtained.

### 2.2 Survey questions

We analyzed qualitative data from the Global Report Survey for the following two open-ended questions. Participants were asked: “Write down anything that has made it harder to keep safe and cope during the pandemic. Think about yourself and everyone in your home when answering.”, and “Write down anything that has made it easier to keep safe and cope during the pandemic. Think about yourself and everyone in your home when answering.” The survey provided participants with a text box without a character limit to respond.

### 2.3 Ethics approval

This study was approved by the Research Ethics Office (Institutional Review Board) of the Faculty of Medicine and Health Sciences at McGill University (study ID: A10-M75-12B).

### 2.4 Data validation

A two-stage screening process was used for data validation. Once the survey closed, the dataset (*n* = 2,133) was verified for invalid cases. In the first stage, the research team identified potentially erroneous and invalid responses by checking for: (a) duplicate IP addresses, (b) incorrect responses in free-text fields (e.g., participant's name), (c) duplicate responses to open-ended questions, (d) completing the survey in less than ten minutes, (e) impossible time gap between the ages of caregiver and the respondent, and (f) cases where the same multiple-choice response was selected repeatedly. Data were cleaned and responses from 883 caregivers of children and youth with disabilities were deemed valid. Responses from participants for this project were retained if they responded to both open-ended questions selected.

### 2.5 Data analysis

Qualitative data from open-ended question responses underwent thematic analysis using inductive coding (61, 62). English and French responses were reviewed and coded by the bilingual lead author (AK), trained by a supervisor and senior trainee (KS, SY). Codes were defined as labels assigned to text from the open-ended responses of caregivers.

We used NVivo software (version 1.7.1) to store and organize qualitative data and codes. Open-ended question responses were uploaded to NVivo software with their numerical participant identifier to record and link the respondent's province of residence and other sociodemographic information.

The dataset underwent two rounds of coding to account for coding errors. A codebook was created containing codes emerging from the dataset and start and end dates for coding were recorded. Definitions for each code were drafted and included. The codes were then reviewed and grouped into overarching codes, based on common themes. For example, the codes “mental health complications in the youth with a disability” and “mental health complications in the caregiver” were grouped into the “mental health” overarching code. Overarching codes were identical for both barriers and facilitators. The codes’ definitions were consulted when collapsing and expanding codes in subsequent analysis phases. The lead author (AK) met regularly with the supervisors (ME, KS) and a senior trainee (SY) to review codes, discuss analysis, and make decisions in group about collapsing, expanding, and new directions. Any changes made to the codebook (e.g., merging of two similar codes, removing duplicate codes, etc.) were dated and initialed on a record sheet.

### 2.6 Researcher positionality

Researcher positionality refers to an individual's world view and the position they adopt about a research task and its social and political context (63, 64). Qualitative researchers often disclose their social location with respect to their areas of focus, with some suggesting that a scientist's proximal positionality to their area of focus often strengthens their analysis (64).

In undertaking research focused on factors related to coping among caregivers of youth with DDDs during the pandemic, it is essential to acknowledge and articulate the lead author and analyst's (AK) positionality. The lead author is a primary caregiver to an autistic young adult who possesses complex needs and requires ongoing support. Their roles as a caregiver and a researcher bring distinctive perspective to this study, influencing the way they approached, interpreted, and contextualized participants’ experiences.

The lead author possesses an in-depth understanding of challenges faced by caregivers of youth with DDDs, both within and beyond the context of the COVID-19 pandemic. This perspective enabled a heightened sense of empathy and comprehension evident in the interpretation and analysis of participant responses. It is nonetheless necessary to acknowledge the potential for subjective emphasis on certain aspects of participants’ experiences and inadvertent oversight of others. To address this position, a reflexive approach was maintained throughout the research process, involving ongoing self-examination of assumptions and pursuit of alternative viewpoints in collaboration with other members of the research team (KS, SY, ME).

## 3 Results

A total of five hundred and seventy-six (*N* = 576) caregivers from 10/13 Canadian provinces and territories provided responses to open-ended survey questions (see Table 1). Respondents resided in diverse geographic locations including urban, suburban, and rural settings, with both low- and high-income households represented. The children and youth of participants had at least one DDD, but were reported to have multiple diagnoses, with a diverse range of diagnoses represented, including autism spectrum disorder, intellectual disabilities, anxiety disorders, vision and hearing impairments or issues, troubles with mobility, sleeping disorders, eating disorders, chronic breathing problems, and epilepsy, among others.

**Table 1:** Survey respondents by province/territory of residence.

Province	Number of participants (*n *= 576)
Ontario	197 (34.2%)
Quebec	115 (20.0%)
British Columbia	88 (15.3%)
Alberta	79 (13.7%)
Saskatchewan	29 (5.0%)
Manitoba	27 (4.7%)
Newfoundland and Labrador	11 (1.9%)
Yukon	9 (1.6%)
Nova Scotia	8 (1.4%)
Northwest Territories	1 (0.2%)
Unknown/not specified by respondent	12 (1.9%)

### 3.1 Participant characteristics

Table 1 describes the Canadian province or territory of residence as indicated by the respondent. Most participants indicated their province of residence as Ontario, Quebec, or British Columbia. Table 2 describes sociodemographic characteristics for the sample, and Table 3 provides conditions that the child or youth with a DDD has been diagnosed with.

**Table 2:** Sociodemographic characteristics of the respondents.

Sociodemographic characteristics of caregivers of youth with DDDs	Number of participants (*n *= 576)
Language of survey completed by participant	
English	465 (80.7%)
French	111 (19.3%)
Gender identity of participant	
Male	141 (24.5%)
Female	426 (74.0%)
Missing	9 (1.6%)
Gender identity of youth with DDD	
Male	350 (60.8%)
Female	216 (37.5%)
Missing	10 (1.7%)
Age of participant	
11–20 years	1 (0.2%)
21–30 years	61 (10.6%)
31–40 years	339 (58.9%)
41–50 years	116 (20.1%)
51–60 years	39 (6.8%)
61–70 years	9 (1.6%)
71–80 years	1 (0.2%)
Missing	10 (1.7%)
Racial identity of participant	
Indigenous	123 (21.4%)
White/Caucasian	371 (64.4%)
Chinese	4 (0.7%)
South Asian (e.g., East Indian, Pakistani, Punjabi, Sri Lankan)	24 (4.2%)
Black (e.g., African, Haitian, Jamaican, Somali)	17 (3%)
Arab/West Asian (e.g., Armenian, Egyptian, Iranian, Lebanese, Moroccan)	11 (1.9%)
Southeast Asian (e.g., Cambodian, Indonesian, Laotian, Vietnamese)	3 (0.5%)
Filipino	3 (0.5%)
Latin American	11 (1.9%)
Korean	1 (0.2%)
Other	14 (2.4%)
Missing	8 (1.4%)
Educational attainment of participant	
Elementary school or less	2 (0.3%)
High school	56 (9.7%)
Diploma	129 (22.4%)
Undergraduate degree	233 (40.5%)
Master's degree	96 (16.7%)
Degree in Medicine, Dentistry, Veterinary Medicine, Optometry, or Law	16 (3%)
Doctorate	5 (0.9%)
Other	23 (4%)
Missing	16 (2.8%)
Household income in 2019	
Less than $20,000	38 (6.6%)
$20,000–$39,999	55 (9.5%)
$40,000–$59,999	135 (23.4%)
$60,000–$79,999	136 (23.6%)
$80,000–$99,999	106 (18.4%)
$100,000–$250,000	86 (14.9%)
>$250,000	6 (1%)
Missing	14 (2.4%)

**Table 3:** Conditions the child or youth has been diagnosed with.

Conditions the child or youth has been diagnosed with	Number of participants (*n *= 576)
Autism spectrum disorder (ASD)	228 (40%)
Intellectual disability (ID)	209 (36.3%)
Anxiety (e.g., generalized, separation, social)	141 (24.5%)
Depression	58 (10.1%)
Epilepsy	115 (20%)
Seizures	74 (12.8%)
Allergies	69 (12%)
Vision/hearing problems (e.g., blindness, deafness, sensitivity to certain lights/sounds)	113 (19.6%)
Troubles with mobility (e.g., cannot walk, difficulty walking or climbing stairs, limping)	127 (22%)
Chronic breathing difficulties (e.g., wheezing, shortness of breath)	47 (8.2%)
Gastrointestinal difficulties (e.g., problems digesting food, constipation, diarrhea)	86 (14.9%)
Eating disorder (e.g., problems eating or swallowing food, lack of appetite)	79 (13.7%)
Sleep disorders (e.g., insomnia, night terrors)	120 (20.8%)
Other	100 (17.4%)

### 3.2 Thematic analysis

Codes were assigned to open-ended question responses from caregivers of the survey. These codes were then grouped into 12 overarching codes based on their themes. Table 4 contains a list of the preliminary overarching codes emerging from the dataset.

**Table 4:** Overarching code definitions.

Overarching code	Description
Child's condition	Caregivers described factors related to coping with the pandemic that are associated with the disability/condition that their child/youth possesses.
Mental health	Caregivers described factors related to coping with the pandemic that are associated with the mental health of themselves, their youth with a disability, and the rest of their families.
Physical health	Caregivers described factors related to coping with the pandemic that are associated with the physical health of themselves, their youth with a disability, and the rest of their families.
Caregiving	Caregivers described factors related to coping with the pandemic that are associated with caregiving for their youth with a disability and maintaining their families’ wellbeing.
Relationships	Caregivers described factors related to coping with the pandemic that are associated with their interpersonal relationships along with the relationships in their homes.
Family finances	Caregivers described factors related to coping with the pandemic that are associated with their ability to generate and maintain their household income.
Education	Caregivers described factors related to coping with the pandemic associated with educational institutions and services.
Public services	Caregivers described factors related to coping with the pandemic associated with public social services or their governments.
Community	Caregivers described factors related to coping with the pandemic associated with interactions and resources from their local communities.
Environment	Caregivers described factors related to coping with the pandemic associated with their physical environments (e.g., indoor, and outdoor spaces).
Information	Caregivers described factors related to coping with the pandemic associated with information made available via various sources of media (e.g., press conferences, safety guidelines regarding COVID-19).
COVID-19	Caregivers described factors related to coping with the pandemic that are specific to COVID-19 (e.g., the virus itself, public health measures and restrictions).

#### 3.2.1 Caregiver-identified barriers to coping with the pandemic

Caregivers described what made coping difficult during the pandemic. Barriers to coping at the individual level are described below and included: (a) mental health complications experienced by the caregiver, their child or youth with a DDD, and other members of the family; (b) caregiver fatigue; (c) maintenance of physical health and sanitary measures; (d) limited access to health and social services; (e) disruption of education services; (f) caregiver employment challenges and situations.

##### 3.2.1.1 Mental health complications

Mental health complications for the youth with a DDD were identified as a perceived increase in anxiety and depression since the beginning of the pandemic, dysregulation, and distressed behaviours related to the youth's disability (e.g., increase in frequency of meltdowns, acts of aggression and violence against themselves or others within the home, refusing food and/or sleep). A caregiver from Ontario reported: “*It has been hard to cope because my children thrive on routine and I’m not able to provide it for them. Because of this, they have regressed in every possible way. [They engage in] self-injury […] and [are] violent [toward us]. [They are] no longer sleeping at night and [refuse] food […]. My husband is working full-time [until] midnight, so I don't get a break from the children, and we are all just emotionally drained and exhausted.*” (ID: 60) Another caregiver from Quebec explained: “*The most difficult thing was the drastic change in my son's routine. Routine changes are very difficult for him and going from regular trips to school [and] respite to sitting at home all day was extremely difficult for him. He had a lot of aggression and anxiety because he didn't understand what was happening.*” (ID: 394)

In contrast, mental health complications mentioned in caregivers were perceived increases in anxiety and stress, lack of sleep, feelings of depression, hopelessness, and loneliness, and heightened familial and/or marital conflict. A respondent from Alberta described: “*No time for myself: playmate, parent, [work] and homeschooling. Not a minute to do anything for me. Arguing with my husband. No control for the present or future causes incredible anxiety. My child with autism needs to move and be out and about. He's going crazy, anxiety is up. We have no choice but to stay home. It's so hard. Worried about the impact on my daughter as well. Worry. Worry. Worry. All. The. Time.*” (ID: 88) Another caregiver from Ontario expressed: “*I was at a loss what to do with my life.*” (ID: 48) Some caregivers also articulated a perceived increase in the frequency of events of domestic abuse. A sibling caregiver from Quebec stated: “*[…] Since the beginning of [the pandemic], I experienced violence in my home and had to live with constant fighting throughout the entire pandemic, since there was no way to avoid the person in question without being able to go to work or school.*” (Translated from French; ID: 249)

##### 3.2.1.2 Caregiver fatigue

Survey respondents also expanded upon caregiver fatigue as a stressor, with one caregiver from Alberta stating: “*I found it difficult to get a break. Ideally, I wanted them out of my home while I tidy. This was not possible. My [expletive] husband was still doing his masters and was reluctant to take them out. All childcare and education [were] dumped on me. I am a healthcare worker and [have] to find my own time to learn more about the virus. […] I suffer. Like most women. Right?*” (ID: 97) Another individual-level barrier was having more than one child with a disability. Caregivers spoke to the difficulty of listing and then prioritizing their family members’ and their own competing needs. A caregiver from Alberta expressed: “*One child finds video conferencing too stimulating and [cannot] participate without being dysregulated for the remainder of the day. The other child is severely dyslexic and needs help to do even basic homework which isn't possible to give while trying to work from home.*” (ID: 78) Several caregivers also reported a lack of or completely restricted access to psychological services as a barrier to coping related to mental health for the whole family.

##### 3.2.1.3 Maintenance of physical health and sanitary measures

Another obstacle faced by these families revolved around the management of physical health and hygiene during quarantine. Participants confirmed the restricted access or complete unavailability of family and specialist physicians, physical, speech, and occupational therapy services, and prescription medication. A caregiver from Ontario explained: “*The limit on prescriptions has made it challenging to venture out monthly as a single parent with a child in a wheelchair [who is] immune compromised. Hard to keep safe when some hospital appointments weren't rescheduled, and we still needed to make it to them.*” (ID: 137) Similarly, another caregiver from Quebec stated: “*My daughter had intense tooth pain during quarantine and finding a dentist during quarantine [was difficult], (even more so for a 25-year-old with a severe intellectual disability) and normally it's hard.*” (Translated from French; ID: 338) Moreover, several participants reported difficulties with following public health measures in place to reduce the spread of the virus, such as social distancing, mask wearing, and frequent handwashing. A caregiver from Alberta reported: “*My daughter has very limited [spatial] awareness so this hampers her physical distancing, and it also makes strangers upset because they do not understand. She has sensory issues and cannot wear a mask.*” (ID: 89)

##### 3.2.1.4 Limited access to health and social services

Caregivers described the limited or no access to public and private social and health service-related supports for themselves or their child with a disability as a barrier to coping with the pandemic. This included hindered access to autonomous and semi-autonomous living centres, in-home and out-of-home respite care services, and rehabilitation services, among others. The moratorium of such services was reported as resulting from lockdowns related to virus outbreaks and understaffing. One participant from Alberta spoke to this loss of services: “*Lack of support. Zero respite. Zero. That's how it is for every parent of a disabled child. For those who are single parents, their emotional [and] mental health [are] deteriorating. There is zero help. Zero.*” (ID: 99) Some caregivers also expressed that some health- and social-related services that had transitioned to online platforms, such as Zoom and Skype, were rendered ineffective, as maintaining the sustained attention of their youth for the duration of the online session was unfeasible.

Caregivers highlighted the moratorium of in-home services as a significant barrier to coping, such as aid from Personal Support Workers (PSWs). One caregiver from Ontario stated: “*Access to nursing and PSW supports is limited as staff also work out in the community. [Ontario Local Health Integration Networks] staff that work for families* via *self-directed funding were not offered the 4$ top-up for front line workers until [two] days ago. This meant many nurses and PSWs choose to work out in the community instead of [committing] to working with one family.*” (ID: 433) Another reported issue for access was the inability for caregivers to see their special needs child or young adult that was staying in a public residence due to lockdowns. A caregiver from Quebec stated: “*My daughter lives in [publicly funded] intermediate resource [housing] and I was not allowed to visit her. This caused me great distress and my daughter had to increase her prescription medication.*” (Translated from French; ID: 275)

Finally, several caregivers reported feeling a lack of access to administrative staff of their health and social service providers, along with a lack of care coordination for their youth with a DDD. A caregiver from Quebec reported: “*We were alone in taking care of our autistic child along with our four-year-old daughter, all while working from home. We asked for help from our CLSC [Centre local de services communautaires] and we are still waiting for a response. While looking for help, we are bounced around from one person to another, and this becomes extremely exhausting.*” (Translated from French; ID: 277)

##### 3.2.1.5 Disruption of education services

Caregivers also spoke to difficulties related to their child or youth's education and schooling, namely the loss of access to teachers and academic staff and navigating the transition to online schooling. Several participants noted a limited ability to keep up with learning materials and contend with other caregiving, work, and academic responsibilities. One caregiver reported: “*My child is in shared custody with [their] father. Taking care of a child by myself with non-verbal autism while working on a major project for work in a university setting was very stressful. I could not provide educational support and enough physical activity. Just handling basic needs was overwhelming. […]*” (ID: 247) Another caregiver from Alberta expressed “*[Not having] school has impacted us. Change in routine to online school has been very hard. [Implementing] a new schedule has been next to impossible. [To] support my autistic child, I would need help or more hours in a day to be prepared for the next [day's] activities, assignments, and schedule. […] Missing friends, teachers, family, and routines. New rules to learn. Some [too] hard to understand with a receptive language delay. […]*” (ID: 138) Other barriers to coping that were related to schooling included challenges with sustained attention for their youth and online classes, reported cuts to the schools’ budgets, and a lack of material resources needed for online learning, such as a stable internet connection and computers.

Several caregivers also reported the halted educational development and progress of their youth with a disability as a barrier to coping. One such caregiver reported: “*We had the most amazing and supportive teacher this year and our child was finally making progress on his social skills. He was on the precipice of positive change. And all of that has been wiped out. Next fall, if school returns, he will be a shell […] of himself, afraid to be close to people. Also, his time with this amazing teacher is finished and we have to learn and connect with yet another one.*” (ID: 246) Another caregiver from Ontario explained: “*The sudden disconnect from school supports was tough. It took a while to get the teachers able to connect directly with students. Online learning worked for our [neurotypical] child, but not for our child with additional needs. He needs 1:1 support to access the curriculum. With both of us, [his parents], working full time from home, there was no one extra to support the work that was sent home.*” (ID: 434)

##### 3.2.1.6 Caregiver employment challenges and situations

Another barrier to coping with the pandemic included job loss, a reduction in working hours, unemployment, and an overall worsening of the family's finances. Some reported struggling to pay for food and living essentials, along with housing and other living costs. A caregiver from Quebec reported: “*I have worked in a specialized school for 22 years. I won't be able to return to work because I do not have the financial means to pay someone to [watch my 27-year-old daughter] for 38 h per week. [This means we are living with] financial insecurity, job loss without access to [Canada's Emergency Response Benefit] CERB, a significant decrease in revenue for the household, and enormous stress.*” (Translated from French; ID: 330) Another caregiver from Quebec described “*[…] my employer decided to no longer provide the option of working from home and forced me to take a leave without pay because he did not consider my child's condition. […]*” (ID: 319) (Translated from French) Moreover, several caregivers reported being an essential worker during the pandemic as a barrier to coping, with some displaying concern about bringing the virus into their homes and a lack of protective materials against the virus provided by public employers.

External barriers that related to their ability to cope are described below and included: (a) limited access to play and physical activity; (b) limited social interactions, and (c) lack of accessible information and supports.

##### 3.2.1.7 Limited access to play and physical activity

External barriers to coping with the pandemic included insufficient outdoor and indoor spaces for play and leisure for the youth and their caregiver. A caregiver from Ontario stated: “*We have a backyard for our daughter to play, however not having access to playgrounds has been very hard as she is a very active child who needs to burn off energy to be happy.*” (ID: 399) Moreover, several caregivers reported small living spaces as a barrier to coping with the pandemic, with a participant from Quebec describing: “*It is extremely difficult for six people to be in a 5 and a half [apartment] constantly.*” (Translated from French; ID: 249)

##### 3.2.1.8 Decreased social interactions

Other barriers that were specifically related to the COVID-19 pandemic included a significant decrease in social interactions with people living outside the home due to mandatory lockdowns and a general fear of contracting the virus. This fear of contracting COVID-19 was sometimes reported to be exacerbated when either the child or the caregiver was immunocompromised or an essential worker (e.g., physicians, nurses, teachers, etc.). One such caregiver from Quebec described: “*I needed to leave the home for work, and despite the measures we took [to avoid virus contraction], we received a false alarm. This worried me terribly because I do not want my daughter to contract the virus. I couldn't imagine her being alone in the hospital.*” (Translated from French; ID: 316) Other external stressors reported by participants included a dearth of essential and protective materials (e.g., masks, surgical gloves, disinfectants) and cleaning supplies. This stressor became particularly challenging when reported by families living in rural areas. One caregiver from Ontario stated: “*Living in a rural environment means driving everywhere. It was often tricky to get to places before [they] closed. It was tricky to get to places before they sold out of some cleaning supplies.*” (ID: 29)

##### 3.2.1.9 Lack of accessible information and supports

Moreover, several participants reported feeling abandoned by their governments. One such caregiver from Ontario stated “*[…] We were again forgotten by our [government] so we struggled with adding expenses and reduced [working hours] for my partner.”* (ID: 115) Several caregivers also reported that managing information from various sources was a barrier to coping with the pandemic. Specifically, some participants reported receiving inconsistent or too much information regarding the state of the spread of the virus and how to mitigate its spread. A caregiver from Quebec reported: “*Trying to act and live like everything is normal but obviously it's not [made it harder to cope]. Also, the day-to-day changes in information we have received from the [government] and school has made it difficult to keep up with what is going on.*” (ID: 52)

Finally, several caregivers also reported a lack of information specific to the needs and risks of COVID-19 infection of children and youth with disabilities as a barrier to coping. One such caregiver from Quebec stated: “*There is a lack of communication between [my son's] healthcare team to know whether our son had specific risks with this novel virus.*” (Translated from French; ID: 273) Some caregivers also reported feeling alienated by their governments, citing a lack of communication of their needs. One such caregiver from Manitoba stated: “*Families like ours were absolutely ABSENT from any conversation from politicians. We had no security for our daughter if me or my husband would be symptomatic. This felt like life or death.*” (ID: 401) A participant from Alberta also described: “*The media saying only the vulnerable are at risk making it sound like our kids do not matter in this.*” (ID: 130)

#### 3.2.2 Caregiver-identified facilitators to coping with the pandemic

Several caregivers reported that nothing made coping and keeping safe during the pandemic easier. For instance, a caregiver from Alberta reported that “*Nothing [has helped]. It has been horrible.*” (ID: 69) Other caregivers reported some facilitators, but reiterated barriers to coping. One such participant from Manitoba stated: “*Staying home, online ordering, and closures made it easier to stay safe, even [though] at the same time those same things made it harder to cope.*” (ID: 390)

Individual-level facilitators related to coping during the pandemic were identified by caregivers as: (a) leisure; (b) calmer daily routines; (c) access to health and education services; (d) support from extended family and community; (e) maintenance of basic organization structures; (f) financial supports, (g) access to protective measures.

##### 3.2.2.1 Leisure

Leisure time, including media entertainment (e.g., watching movies and television shows and scrolling through social media platforms) and engaging in hobbies were facilitators in coping with the pandemic. Engaging in hobbies such as baking, home decorating, painting, meditation, and gardening were perceived as positive, as well as increased opportunities for time spent together. A caregiver from Manitoba stated: “*Our family is great comfort for each other. We've relished our time home together. More family time, more outside time and more healthy practices.*” (ID: 486) Another example of uplifting family interactions was videoconferencing with extended family (e.g., grandparents, aunts, and uncles, etc.) through platforms like Zoom and FaceTime. This was described as a facilitator to coping in both the youth with a DDD and the caregiver.

Facilitators linked to physical wellbeing included engaging in exercise for both the caregiver and the youth with a DDD, when possible, adopting a healthy diet, and staying adequately hydrated throughout the day. A caregiver from Quebec shared: “*Finding thirty minutes to exercise on my elliptical, going for a walk by myself! Eating well.*” (ID: 88) Another caregiver from Alberta expressed: “*We are lucky to have a backyard and a nature/green space at the end of our street that no one else seemed to visit which allowed the kids to easily get out of the house and get some fresh air and exercise.*” (ID: 133) Caregivers also highlighted the importance of sustained access to outdoor spaces for leisure and sports as a means of coping with the pandemic, with some noting that living in a rural area facilitated this. One participant from Manitoba mentioned: “*living in a rural location where we could still play outside without interacting with other people.*” (ID: 421)

##### 3.2.2.2 Calmer daily routines

Several caregivers also described perceived decreases in anxiety related to changes in their youth with a DDD. One such caregiver from Alberta described: “*Limited transitions means fewer transition tantrums.*” (ID: 89) Another caregiver from British Columbia expressed: “*There is less interaction with society. Less driving in traffic, less being in crowded places. This makes it easier and less mentally draining. My children are not completely drained after the end of the school day and having meltdowns.*” (ID: 139) Caregivers also spoke to the ability of their child to understand the pandemic as being a factor that facilitated their own ability to cope, with several caregivers citing child skill improvement in lockdown. Several caregivers also spoke to allowing their child to develop their own coping skills, with a participant from Ontario reporting that “*[…] Letting my son dictate how much and what he does. Letting him have more screen time if that keeps him more […] balanced emotionally.*” (ID: 412)

Another facilitator impacting family coping consisted of a perceived “slowing down” of life. This perceived slowdown was described as no longer needing to commute to and from work or school while ensuring all family members were prepared for the day, with some participants even reporting being retired or on maternity leave as a facilitator to coping. A caregiver from Quebec stated: “*Less stress due to no longer needing to wake up early and rush to get ready for school, more sleep, had time to play outside and walk each day. I was receiving an income so no financial stress, I could help my children with their academic tasks and see where they were at, family game time, having internet, we could stay in touch with parents and friends.*” (Translated from French; ID: 353) Several caregivers also reported the implementation of a new routine as a facilitator to coping. In addition, some participants cited finding ways to connect to routines implemented prior to the start of the pandemic and its subsequent quarantines. One such caregiver from Quebec described: “*Taking [my son] for daily drives to see his favourite places helped a lot.*” (ID: 91) Some caregivers also reported setting up a contingency plan for quarantining and caregiving among members of the home should they contract the COVID-19 virus as a facilitator to coping.

##### 3.2.2.3 Access to health and education services

Caregivers reported maintained access to healthcare providers, whether through virtual means such as videoconferencing and telephone calls or in-person appointments, as a facilitator to coping with the pandemic. A caregiver from Ontario described: “*Virtual medical appointments made it easy to keep up with our health. Travel and doctor visits are stressful for my child, being able to do these from home, saved hours of stress on our family.*” (ID: 433) Several caregivers cited pre-existing experience and expertise in navigating public health- and education-related systems and supports as a facilitator to coping.

Maintained access to teachers and educational staff was also reported as a facilitator by caregivers, as was a solid understanding of navigating the educational system during the pandemic and an awareness of scholastic resources available. A caregiver from Quebec stated “*I have great knowledge of the educational field, which facilitated homeschooling. My children cooperated well and with a family meeting, we implemented a routine that responded to everyone's needs.*” (ID: 349) (Translated from French). Another participant from Ontario reported that “*It does make it easier when the educators […] provide [my son with] sensory items and resources to try and keep him happy. The school has been essential to me in trying to get my children back on track.*” (ID: 60)

##### 3.2.2.4 Support from extended family and community

Receiving caregiving support from other family members, friends, and community organizations was also identified as a facilitator for coping. This manifested in various ways, encompassing caregiving assistance such as respite and in-home care providers, alongside community and online resources, and even general check-ins (e.g., online educational materials, virtual peer and parent support groups, community centers sending care workers for check-ins to families’ homes). One caregiver from Ontario explained that “*[they] have wonderful neighbours who allow [their] children to ride their bikes down their trails […], a neighbour who brings [them] food and helps weed the garden. [The] local [Communities That Care Centre] has an excellent parent support group and many caring parents ([and] a couple staff) that help brainstorm ideas.*” (ID: 398) Furthermore, several participants highlighted the sense of community and witnessing compassion in others as additional facilitators for coping. A caregiver from Yukon reported, “*The grocery stores in town have really gone above and beyond to make sure folks can continue to shop. That's because the owners care […].*” (ID: 372)

##### 3.2.2.5 Maintenance of basic organization structures

Some caregivers pointed to the maintenance of the cleanliness and organization of the family home and spaces as a facilitator to coping. Several caregivers also described attributing work- and school-related functions to specific areas of their homes as an organizational facilitator related to coping. For instance, a participant from Alberta stated: “*Setting my husband's work area up separately in the basement helped him to stay working.*” (ID: 119) Additionally, maintained access to food and grocery delivery services and online shopping for essentials, such as disinfectant products, laundry products, and personal hygiene products, was described as a facilitator to coping during the pandemic. Delivery of such products was also explained as a facilitator to coping, as caregivers felt safer when they did not need to enter stores to purchase essential items where they were at risk of contracting the virus.

##### 3.2.2.6 Financial supports

Caregivers also reported facilitators to coping that were related to household income. Having the option of working from home was a facilitator to coping, in that they were able to supervise their youth with DDDs during school closures, along with maintaining a stable financial income. Several participants also reported that public financial aid helped them to cope. Specifically, the Canada Emergency Response Benefit (CERB) provided financial support to any employed and self-employed Canadian citizens whose employment status was directly affected by the COVID-19 pandemic, with eligible recipients receiving CAD$2,000 for each four-week period they required, between March 2020 to May 2022. A recipient of CERB from Quebec stated: “*The Canadian Emergency Response Benefit […] has been a huge relief because with that I don't need to do Uber (my second job), so I have more time to take care of my son.*” (ID: 511) Several caregivers reported finding ways to save money as a facilitator to coping.

##### 3.2.2.7 Access to protective measures

Facilitators specifically related to the COVID-19 pandemic involved following public health measures, such as wearing a mask, social distancing, and isolation, when possible, for all members of the family. Several caregivers also reported having access to protective and disinfectant materials as a facilitator to coping and feeling protected from the virus. Moreover, caregivers reported that receiving information and updates on the spread of the virus from governmental representatives and experts, along with Health Canada's recommendations on staying safe, facilitated their coping.

### 3.3 Caregiver perspectives and alignment with policies

Several of our team members (AK, SY, KS, ME) participated in a collaborative effort to analyze Canadian policies during the initial phases of the COVID-19 pandemic (65). This encompassed an investigation of policies published by provincial and territorial governments from September 2020 to April 2021, marking the pandemic's initial stages. Policies included for analysis pertained to the pandemic specifically, referred to persons with disabilities or their caregivers, and included youth (<24 years). Our approach involved employing text mining techniques in conjunction with thematic analysis to assess policy content while focusing on their alignment with UNCRPD articles and mental health supports. Specifically, our team developed a mental health categorization model specifically addressing mental health objectives (65). We operationalized mental health according to the WHO's definition in relation to the COVID-19 pandemic and assessed policies using this mental health impact model (65).

Employing our mental health impact model, our analysis revealed a relatively restricted scope of policies addressing the psychological implications of the pandemic on youth with DDDs (65). Some policies acknowledged potential mental health risks stemming from disruptions to daily routines and prolonged lockdown-related isolations. The subset of policies highlighting these risks was nonetheless even smaller when it came to addressing unique needs of youth with DDDs and their families. Additionally, none of these policies proposed action plans featuring specific services aimed at mitigating adverse effects or fostering mental wellbeing during or after the pandemic for this group (65).

When considering the analysis of provincial policies published during the pandemic's initial stages (65), evidence from the present study underscores a discernible misalignment between caregiver-identified needs and public supports available during a national emergency. Notably, a limited number of COVID-19 policies were effectively aligned with practical services that addressed caregiver-identified barriers, such as extending assistance for mental health complications and ensuring ongoing service access for youth with DDDs. For instance, schools were considered to be the main community setting for children in the policies included for analysis. A subset of provincial policies related to education for youth with disabilities acknowledged the role that schools perform in providing daily essential services for this group, however concrete implementation mechanisms to address issues related to service losses were scant (65). Difficulties related to financial instability during the pandemic were also a notable barrier. While broad financial supports were made available federally, such as CERB, our analysis captured a very limited number of provincial financial assistance specific to youth with DDDs and their families. Some policies only providing financial resources to families to cover some extra costs for caring for their child with a severe disability (65).

Our results also indicate that facilitators did align with pre-existing policies. Caregivers reported access to play and leisure and outdoor spaces, along with physical activities and sports as facilitators to coping. However, many COVID-19 public health restrictions were deterrents to accessing these facilitators as policies during the pandemic. A growing concern thus revolves around the inadequacy of considering social determinants of health in policymaking for youth with disabilities during and outside the context of emergencies (66), with this issue only amplified by the context of the pandemic.

## 4 Discussion

The objective of this project was to describe caregiver-identified barriers and facilitators related to their coping during the COVID-19 pandemic. Our aim was to contextualize the experiences of youth with DDDs and their families during this public health emergency while describing their alignment with Canadian public policies targeted at this demographic. The outcomes of our study reveal that this global disastrous event impacted the mental wellbeing and external stressors faced by youth with DDDs and their caregivers.

Our thematic analysis found that caregiver-identified coping factors aligned with existing literature. Caregivers articulated perceptions of negative mental health impacts for both them and their youth with a DDD, consistent with emergent COVID-19 findings pertinent to this demographic (4, 41). Participants further communicated a depletion of services and constrained care coordination between their youth's healthcare providers, also mirroring trends in existing literature (54, 67). Stressors encompassing physical health challenges during the pandemic, including gaps related to telehealth and services accessed through educational systems, have also been previously documented within this group (68–70). Moreover, several coping facilitators may support modifiable factors for resilience, such as parent self-efficacy (56), as reported by caregivers in our sample. This includes receiving support in accessing schooling with online and material resources, maintained telehealth and in-person services and interventions where possible, and public financial supports in the form of tax credits and emergency benefits. Some caregivers also reported improvements in their youth's skills (e.g., self-regulation, motor skills) as a facilitator to coping during the pandemic, which is consistent with pre-existing quantitative data from the same sample (57).

Exploring the alignment between caregiver-identified barriers and facilitators to coping during the COVID-19 pandemic and Canada's social policy landscape reveals a significant gap in the way policies respond to the most pressing needs of families of children with disabilities. The WHO *Global Report on Developmental Disabilities and Delays* initiative, aimed at documenting the experiences of youth with DDDs and their families throughout a global disastrous event, provides an overview of how families across the globe experienced this worldwide public health phenomena. Our work holds distinctive value as it stands as the inaugural endeavor to delve into the *Global Report Survey*'s substantial qualitative dataset, thereby enhancing the depth of insights derived from this valuable resource, namely with respect to policy analyses developed by the same team. It thus expands the thinking of intersections of health and policy, with the understanding of how policies reflect human rights frameworks and protect equity-deserving groups.

Social determinants of health can be described as non-medical factors that have been found to exert influence on health outcomes, encompassing facets such as income, education, unemployment and job security, housing, and food insecurity, social inclusion and discrimination, among others (71). Disability is considered one social determinant of health (72, 73), and intersects with many others, justifying further the use of normative frameworks as the UNCRPD to guide policy and program development for this population. This framework considers many aspects that are crucial for wellbeing, including health services, rehabilitation, and community living (74, 75). Within Canada's federalist context, many public services that underpin social determinants of health are offered through educational systems and schools (76–78). These systems faced notable disruptions and service moratoriums during the pandemic, speaking to the vulnerability of maintaining a high reliance on these settings to deliver essential services.

There is also increasing concern in the disability advocacy community about individuals with DDDs and their families and caregivers being insufficiently considered and included by decisionmakers when designing policies and supports for them (66). Our findings indicated that many families of youth with DDDs felt alienated by their governments and public discussions around the needs of persons with disabilities during the pandemic. Improved consultation of persons with disabilities and youths with DDDs and an overall shift toward a policy co-design approach to policymaking for these groups could support the creation of better measures and a lesser sense of neglect in policy and program development (79). Children, youth, and their caregivers and families, along with community organizations and professionals, should be engaged in policy co-design at all stages of policy development, including conceptualization, drafting, and implementation, facilitating policy co-design that can better reflect this population's priorities.

Findings from the current study may be timely, given the recent passing of national strategy legislation for autism spectrum disorder (ASD), the most prevalent neurodevelopmental disability, by the Canadian Parliament. The Bill S-203, *An Act respecting a federal framework on autism spectrum disorder*, received royal assent in Canadian Parliament in March 2023. The Act mandates the drafting of a national framework for autism policy by the federal Ministry of Health. This framework must identify measures to enhance equitable access to screening and diagnosis, financial support for autistic persons and their families, a national research network to promote research and improve data collection, national public knowledge campaigns, accessible and culturally relevant resources on evidence-based information to support autistic persons and their caregivers, and mechanisms to ensure accountability in the use of federal funds (80).

The passing of Bill S-203 and its resulting drafting of a national framework represent a unique and unprecedented opportunity in Canada to integrate evidence-based findings and a rights-based approach into autism policy. The needs of children and youth with DDDs and their families must be considered in emergency planning, as our results reinforce the notion that families experienced marginalization from service acquisition and access during the pandemic. Development of financial benefits and supports, such as potential tax benefits and/or direct financial supports should be considered, such as updating the Canada Disability Tax Credit (81). Considerations include enhancing coordinated care between health, social, and education service providers. Information related to the COVID-19 virus and services and recommendations available must be accessible in language and have plain-language formats.

Finally, our results reinforce the notion that the COVID-19 pandemic exacerbated pre-existing inequities for youths with disabilities and their families. Several caregivers described an alienation from public systems and service infrastructure that was only amplified by this global disaster. When drafting policy for youth with DDDs, it is essential to enshrine human-rights language, as outlined by the UNCRPD, to optimize social determinants of health through public systems and service provision infrastructure and to pave the way for future policy frameworks that align with protecting human rights.

### 4.1 Limitations and future directions

A potential limitation of this study was recruiting from a non-random convenience sample. While a strength of the study is the considerably large sample size of caregivers for the *Global Report Survey* who responded to open-ended questions, our sample may not have been representative of the population of children and youth with disabilities in Canada, particularly those who did not have access to the internet or were not connected to social media or community organizations through which the *Survey* was distributed. Many convenience samples comprise participants that are in proximity or are highly accessible to the research team. In the case of the current study, participants were recruited through our research network's social media networks and mailing lists of partners.

Another potential limitation of this study is that the *Global Report Survey* consisted of a cross-sectional design. These types of study designs offer insight into only one point in time, with a limited ability in describing changes in coping factors over longer periods. The survey was open during the initial weeks of the first summer of the COVID-19 pandemic, offering a snapshot of perspectives of coping during an uncertain period for Canadian families. The school year had just ended, with many children and youth with a DDD not having been in classrooms to receive services they may have been relying upon for over three months. Research about the virus and the way it spread was still scant, with plans for vaccine trials unclear and without a timeline. Travelling was also strongly discouraged by governmental officials, with mandatory two-week quarantines in place for all international travellers (82). In some provinces, some restrictions related to public gatherings (e.g., the reopening of malls), had relaxed, and non-essential travel had reopened (83–85). Moreover, even though there was no specific character limit, responses provided in a written survey format might have been constrained in presenting a comprehensive account of caregivers’ experiences and contexts, as could have been obtained through other qualitative research methods, such as individual interviews.

An examination of how Canadian provinces responded to the COVID-19 pandemic's public health policies revealed inconsistencies. Most public health actions exhibited significant variations in their timing of implementation across different provinces and territories (86). At this juncture in the pandemic, it is conceivable that due to the absence of a definite conclusion to the pandemic and its subsequent protective public health measures, caregivers may have experienced a profound sense of hopelessness, potentially influencing our findings. Consequently, there exists a need for further research to investigate coping mechanisms and mental wellbeing among this population during the later phases of the pandemic and its aftermath.

Moreover, several policies that were included in our discussion on alignment between policy and coping factors were published following the closure of the *Global Report Survey*. It is thus possible that the publishing and implementation of these policies could have affected alignment between caregiver-identified needs during the pandemic and public supports available. The reinstation or addition and implementation of novel public supports during the later stages of the pandemic could have assisted parents who felt inadequately supported by their public services, potentially affecting their responses to our open-ended questions.

Further research should also account for potential differences in responses following postal codes. Barriers and facilitators may have differed for families from rural, suburban, and urban areas, and notably when comparing areas with high vs. low socioeconomic status. Better understanding of variations in such responses can help to tailor supports for these families based on regional supports and public infrastructure available.

## 5 Conclusion

Prioritizing the needs of families of youths with DDDs during a public health emergency can significantly impact their experiences with schooling and mental health. Findings from our study highlighted the need for increasing financial benefits and emergency physical and mental health supports for families of youth with a disability. Maintained offering of telehealth services and creating inclusive public spaces for play are also priority areas for decisionmakers as we emerge from the COVID-19 pandemic. Future legislation around disabilities must enshrine human-rights language, as posited by the UNCRPD, and approaches to promote social determinants of health. Policymakers must develop concrete action plans tailored to a post-COVID Canada for these youths and their caregivers, while enhancing strategies for future emergency planning.

## Data Availability

The datasets for this article are not publicly available due to concerns regarding participant/patient anonymity. Requests to access the datasets should be directed to the corresponding author, KS, keiko.thomas@mcgill.ca.
